# Crisis Intervention Teams as Dual Resources: An Explanatory Sequential Mixed Methods Study of Hospital Staff

**DOI:** 10.1111/nicc.70692

**Published:** 2026-11-10

**Authors:** Sophie Mahn, Jenny Rosendahl, Mona Weiss, Teresa Deffner

**Affiliations:** ^1^ Institute of Work and Organizational Psychology Friedrich‐Schiller University Jena Jena Germany; ^2^ Department of Anaesthesiology and Intensive Care Medicine Jena University Hospital Jena Germany; ^3^ Institute of Psychosocial Medicine, Psychotherapy and Psychooncology Jena University Hospital Jena Germany

**Keywords:** crisis intervention, healthcare staff, hospitals, occupational stress, psychosocial support

## Abstract

**Background:**

Crisis situations in healthcare create substantial emotional and organisational demands for staff. Crisis Intervention Teams (CIT), originally established to support patients and relatives, may also function as organisational resources for healthcare professionals.

**Aim:**

This study aimed to examine whether and how hospital‐based Crisis Intervention Teams (CIT) function as organisational resources for healthcare staff during crisis situations.

**Study Design:**

This study used an explanatory sequential mixed methods design at a large university hospital in Germany. Study I comprised a cross‐sectional survey of physicians, nurses and midwives with crisis experience, comparing staff with prior CIT involvement (‘Requesters’) with those without prior involvement (‘Non‐requesters’) regarding perceived work characteristics and CIT‐related experiences. Study II used focus groups to explain and contextualise the quantitative findings.

**Results/Findings:**

Study I included 70 participants: 48 CIT‐requesters and 22 Non‐requesters. CIT‐requesters reported greater role clarity than Non‐requesters (*U* = 352.00, *p* = 0.025, *r* = 0.27; Hodges–Lehmann difference = 2.00, 95% CI [0.00, 4.00]). CIT exposure significantly interacted with supervisor support, B = −5.23, SE = 2.14, *t* = −2.44, *p* = 0.017, 95% CI [−9.50, −0.95], Δ*R* ^2^ = 0.073; at low supervisor support, non‐requesters reported greater strain. Study II included 12 participants in two profession‐specific focus groups: five physicians and seven nurses/midwives. Qualitative content analysis identified five key themes, including perceived emotional relief and reassurance as benefits of CIT involvement, and limited availability and unclear activation thresholds as barriers to requesting support.

**Conclusions:**

The findings suggest that CIT function as organisational resources for staff. Their impact, however, depends on complementary organisational resources, particularly supervisory support, while restricted availability remains a major structural barrier.

**Relevance to Clinical Practice:**

CIT may reduce emotional strain, strengthen role clarity and help critical care nurses maintain focus on clinical tasks. Clear activation criteria, greater visibility and improved availability may enhance their effectiveness during acute situations.

## Impact Statements

- What is known about the topic
  ○ Healthcare staff in hospitals face high psychosocial strain due to the dual burden of medical and emotional care.
  ○ Crisis Intervention Teams are established in community settings but scarcely implemented or evaluated in hospitals.
  ○ Existing evidence mainly addresses benefits for patients and relatives, not for healthcare providers.
- What this paper adds
  ○ Provides one of the first systematic evaluations of hospital‐based CIT from the staff perspective.
  ○ Shows that CIT are associated with greater role clarity but not with reduced workload or increased autonomy.
  ○ Identifies key barriers to activation (unclear thresholds, limited availability) and highlights staff‐perceived benefits such as emotional relief and reassurance.
  ○ Demonstrates that CIT effectiveness depends on organisational context, especially supervisory support.
  ○ Offers practical implications for embedding CIT sustainably into hospital routines.

## 1 Introduction

### 1.1 Occupational Stress in Healthcare Settings

Healthcare professionals are consistently exposed to high‐stress environments characterised by heavy workload, psychological demands and substantial responsibility [1, 2]. Chronic workplace stress can manifest in physical and psychological conditions and fosters a culture of invulnerability within the medical profession, thereby heightening the risk of burnout and mental health problems such as depression, anxiety and suicidality [3, 4, 5].

Work demands are particularly high in acute, high‐intensity settings such as emergency departments (ED) and intensive care units (ICU), where staff must act under extreme time pressure while simultaneously attending to the emotional needs of patients and families [6, 7]. This combined strain is reflected in the fact that emergency medicine is among the medical specialties with the highest rates of nurse and physician burnout [7, 8]. The dual burden of medical and emotional care jeopardises both employee well‐being and patient safety, through the cumulative effect of stressors [9].

### 1.2 Crisis Intervention Teams as Support Structures

Against this backdrop, prevention strategies for occupational stress are gaining increasing attention in critical care. While individual‐focused interventions—such as stress management—can reduce burnout [5, 10] and healthcare organisations should ensure access to such services, they complement rather than substitute organisational interventions, such as adequate staffing and manageable workloads [11].

It is therefore important to establish organisational‐level strategies that provide structured support, particularly during periods of acute stress. Peer‐support programmes offer direct assistance to employees following adverse medical events [12, 13]. However, they do not address the dual challenge of delivering both medical treatment and emotional care simultaneously.

CIT, by contrast, were primarily established to support patients and relatives [14]. In doing so, CIT may also provide indirect relief for healthcare professionals by intervening in precisely those situations in which this combined burden arises. This indirect relief may be particularly important in ICU and ED settings, given the high number of patient deaths in ICUs [15] and the acute nature of clinical conditions.

Although CIT are well established in community settings in Germany, their systematic implementation within hospitals remains largely confined to these areas [16]. Data on their overall availability and effectiveness in hospitals are scarce [17, 18].

### 1.3 Job Demands‐Resources Model and Psychological First Aid

The potential role of CIT as organisational resources can be further examined through established frameworks like the Job Demands‐Resources (JD‐R) model, which helps to conceptualise how organisational interventions may buffer strain and enhance resilience [19]. The model distinguishes between job demands—physical, psychological, social or organisational aspects requiring sustained effort—and job resources, which support goal attainment, reduce job demands and their costs and foster personal growth [20].

From this perspective, crises represent acute job demands that increase workload through additional emotional and communicative tasks, undermine role clarity by creating uncertainty about responsibilities and restrict autonomy by limiting decision latitude. Although CIT were originally designed for patients and families, they may also serve as organisational resources for staff by buffering these demands [21] through structured communication and by addressing the emotional needs of affected individuals in line with Psychological First Aid approaches [22, 23, 24, 25]. By closely reflecting these principles of immediate, non‐pathologising support [26], CIT can relieve clinical staff by ensuring that psychosocial needs are addressed by trained professionals, while allowing healthcare staff to remain focused on medical care. Despite their growing use, systematic evaluations of crisis intervention teams in hospital settings remain scarce, particularly from the staff perspective. As a result, little is known about whether and how CIT function as organisational resources for healthcare staff. Addressing this gap is essential for quality management, since psychological support interventions cannot be assumed to have uniformly positive effects and therefore require careful evaluation.

### 1.4 Aim

The present study addresses this gap by examining whether and how hospital‐based Crisis Intervention Teams (CIT) function as organisational resources for healthcare staff during crisis situations.

In line with an explanatory sequential mixed methods design, Study I first used a quantitative survey to examine perceived CIT quality and to compare staff with and without CIT exposure regarding workload, autonomy and role clarity. Following the JD‐R model, we expected comparable workload, but greater autonomy and role clarity among employees with CIT experience.

Study II then used qualitative focus group discussions with CIT‐experienced staff to explain and contextualise the survey findings. Specifically, the qualitative phase explored reasons for and barriers to requesting CIT support, perceived effects on staff strain and suggestions for improvement. Together, the integration of both strands aimed to provide a comprehensive understanding of how CIT operate in hospital practice and what this implies for quality management.

## 2 General Design and Methods

### 2.1 Research Design Overview

The study employed an explanatory sequential mixed methods design, consisting of an initial quantitative online survey (Study I) followed by qualitative focus group discussions (Study II) (Figure 1). Study I was prioritised and used to identify patterns in staff experiences with CIT, including perceived work demands and evaluations of CIT support.

**FIGURE 1:**
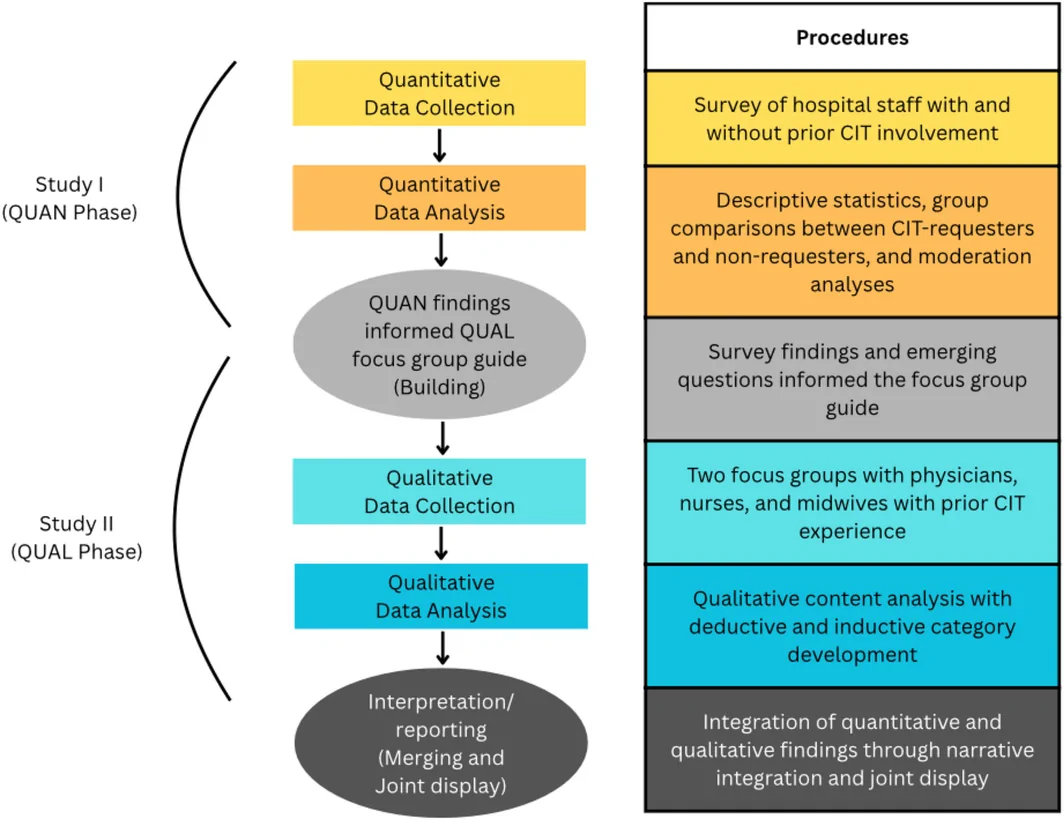
Design overview of the explanatory sequential mixed methods study.

Study II was added iteratively during the research process to address questions that emerged from Study I, particularly regarding mechanisms underlying potential strain reduction, reasons for requesting CIT support, and perceived barriers to utilisation.

This reflects integration through building, whereby results from one phase inform subsequent data collection and is consistent with established explanatory sequential mixed method designs [27].

To enhance transparency and methodological rigour, established mixed methods integration strategies, including building, connecting and merging, were applied [27]. Relevant criteria from the Mixed Methods Appraisal Tool (MMAT; [28]) were used to assess the methodological quality of the quantitative and qualitative components (Appendix S1). Because Study II was added iteratively, the overall mixed‐methods design was not prospectively planned using a mixed‐methods framework. The reporting was subsequently reviewed against the Good Reporting of a Mixed Methods Study (GRAMMS) recommendations (Appendix S2).

### 2.2 Eligibility Criteria

We invited hospital staff involved in direct patient care, including physicians, nurses and midwives from all clinical wards, who had experience with crisis situations. Staff without direct exposure to crises, such as administrative personnel, were excluded. Crises were defined according to Cullberg's [29] model as sudden and distressing events that threaten psychological or social stability.

In Study I, CIT‐requesters were defined as staff members who had either requested CIT support themselves or had been present during a CIT intervention on their ward. Non‐requesters were defined as staff members without prior CIT involvement. In Study II, participants were selected based on prior experience with CIT; the number of CIT interventions they had experienced was not used as a selection criterion.

### 2.3 Ethics Statement

Study I was approved by the institutional ethics committee on 11 April 2023 (Ref. No. 2023‐2974‐Bef) and by the hospital staff council. Study II received separate approval from the institutional ethics committee on 11 June 2024 (Ref. No. 2024‐3394‐Bef) and was formally authorised by the hospital staff council.

## 3 Study I: Survey

### 3.1 Design and Methods

#### 3.1.1 Recruitment and Study Design

Study I used a cross‐sectional, observational design at a large university hospital in Germany. Consistent with the exploratory, field‐based nature of the study, all eligible hospital staff available during the data collection period were invited to participate. Participants were recruited through convenience sampling. Invitations were distributed via posters placed on all wards and through email notifications sent to ward managers and the physicians' mailing list.

The study compared two naturally occurring exposure groups: staff with prior CIT exposure, including those who had requested CIT support themselves or had been present during a CIT response, and staff without prior CIT exposure. These groups are hereafter referred to as ‘CIT‐requesters’ and ‘Non‐requesters’, respectively. In line with the MMAT criteria for quantitative non‐randomised studies, group allocation reflected naturally occurring intervention exposure rather than experimental assignment.

The final sample reflects all respondents who chose to participate within the recruitment period. No formal a priori sample‐size calculation was conducted for the quantitative phase.

#### 3.1.2 Measures

In Study I, the survey assessed sociodemographic and occupational characteristics, perceived work characteristics, potential moderators, reasons for requesting CIT support and structural and procedural aspects of CIT implementation. An overview of all collected variables, respondent groups, and analysis methods is provided in Table S1.

To define the evaluation dimensions for crisis interventions, we drew on established conceptual frameworks within psychosocial acute care and occupational stress research. This process resulted in three overarching evaluation dimensions (Structural and Procedural Aspects, Reasons for Call, Others) with several subcategories (see Table S1). Based on prior research, autonomy, quantitative workload and role clarity were additionally identified as key work‐related stress factors relevant to healthcare staff [8, 30, 31, 32, 33, 34, 35]. These dimensions were selected because CIT support was hypothesised to be associated with staff experiences of these work‐related demands.

To operationalise autonomy, quantitative workload and role clarity, we used the Perceived Work Characteristics Survey (PWCS), a validated instrument with strong psychometric properties in healthcare settings. In the original validation study, its subscales showed good internal consistency, with Cronbach's α ranging from 0.80 to 0.92 across occupations [36]. The PWCS subscales used in this study were translated following the TRAPD model [37].

The present study used 17 PWCS items covering autonomy (six items), quantitative workload (six items) and role clarity (five items). Items were rated on a 5‐point Likert scale ranging from 1 (‘do not agree at all’) to 5 (‘agree strongly’). Subscale scores were calculated by summing the corresponding items, resulting in possible ranges of 6–30 for autonomy and workload and 5–25 for role clarity. The three subscale scores were used separately for the group comparisons. For the moderation analyses, the workload items were reverse‐coded and all three subscales were summed to form a total PWCS score ranging from 17 to 85, with higher scores indicating more favourable perceived work characteristics and lower perceived strain.

To capture structural and procedural aspects of CIT implementation not covered by existing scales, such as punctuality, accessibility and information flow, we included additional study‐specific items. The final survey comprised 29 items, including six open‐ended questions (Appendix S3). Because no standardised instrument for hospital‐based CIT evaluation was available and the study followed an exploratory field design with a limited sample size, psychometric validation of these newly developed items was not feasible at this stage.

#### 3.1.3 Procedure

Data were collected anonymously via the online platform SoSci Survey over a 5‐week period (May–June 2023). Informed consent was obtained electronically; participation was voluntary and uncompensated. Both groups provided demographic information and completed the PWCS‐related items. CIT‐requesters additionally completed the CIT‐specific evaluation items.

#### 3.1.4 Data Analysis

Group comparisons were conducted using the PWCS items completed by both CIT‐requesters and Non‐requesters. Prior to these analyses, the distributional properties of all PWCS subscales were examined using Shapiro–Wilk tests and visual inspection of histograms and Q–Q plots. Workload showed an approximately normal distribution and was therefore analysed using an independent‐samples *t*‐test; this choice was supported by simulation evidence indicating that parametric tests perform robustly with multi‐item Likert scales [38]. Autonomy and role clarity deviated from normality and were analysed using Mann–Whitney *U*‐tests. Significance levels were set at *p* < 0.05.

Four linear regression models tested age, job tenure, supervisor support and colleague support as moderators of the association between CIT exposure and the total PWCS score. Each model included CIT exposure, the respective moderator, and their interaction term. For non‐significant interactions, reduced models without the interaction term were used to estimate main effects. Analyses were conducted using Hayes' PROCESS macro after checking linearity and normality assumptions [39].

### 3.2 Results

#### 3.2.1 Characteristics of the Participants

To provide contextual information on the scale and scope of CIT activity at the hospital, we used aggregated internal CIT records provided by the team. These records documented 191 CIT interventions between November 1, 2020, and July 7, 2023, across multiple units, most frequently the ICU, paediatrics, general wards, the ED, and obstetrics/neonatology. Indications were heterogeneous and included support for relatives following a patient's death, acute deterioration of a child or adult patient, crises associated with childbirth, and assistance with death notifications.

A total of 71 participants completed the survey. One case was excluded because the respondent had no contact with the CIT, as the call had been made outside on‐call hours. The final quantitative sample comprised 70 participants, including 48 CIT‐requesters and 22 Non‐requesters. Overall, 74.3% of participants were female, 68.6% were aged 31–50 years, and more than half had more than 6 years of professional experience (Table 1). Most participants worked in high‐acuity clinical settings, particularly intensive care, paediatric intensive care and emergency care (Table 2).

**TABLE 1:** Characteristics of survey participants (*N* = 70).

Variable	Category	*n* (70)	%
Gender	Female	52	74.3
	Male	18	25.7
Age (years)	21–30	8	11.4
	31–40	24	34.3
	41–50	24	34.3
	> 50	14	20
Tenure in years	0–5	20	28.6
	6–15	24	34.3
	16–25	12	17.1
	26–35	10	14.3
	> 36	4	5.7

**TABLE 2:** Clinical work settings of requesters (multiple responses; *N* = 48; 50 reported settings).

Clinical setting	*n* (48)	%
Intensive care unit (ICU)	18	36.0
Paediatric intensive care unit	10	20.0
Emergency care (EC)	8	16.0
Palliative care	4	8.0
Neonatology	3	6.0
Internal medicine	2	4.0
Paediatric emergency care	1	2.0
Oncology	1	2.0
Paediatric infectious diseases	1	2.0
Labour and delivery	1	2.0
Paediatric surgery	1	2.0

*Note:* One participant reported working in both the Intensive Care Unit and Emergency Care; two participants reported working in both Neonatology and Paediatric Intensive Care, one participant did not provide an answer.

#### 3.2.2 Evaluation of CIT Implementation

Structural and Procedural Aspects. Among the CIT‐requesters included in the analysis, all reported reaching the team on the first attempt and within a timeframe appropriate to the urgency. Further questions focused on on‐call availability, clarity of responsibilities, reliability and effectiveness of information flow. With the exception of on‐call availability, all categories received ratings above the scale midpoint. Assessments of on‐call availability were divided: 47.9% (*n* = 23) considered current hours sufficient, while 52.1% (*n* = 25) rated them as inadequate. Open‐ended responses suggested a preference for extended, ideally 24/7, coverage. Ratings of the remaining dimensions are summarised in Table 3, indicating generally positive evaluations, with minor deductions in clarity of responsibilities and information flow.

**TABLE 3:** Requesters' evaluation of structural and procedural aspects of Crisis Intervention Team (CIT) implementation.

Category	M	SD
Clarity of responsibilities	4.15	0.68
Reliability	4.68	0.78
Effectiveness of information flow	4.53	0.89

*Note:* Ratings were based on a 5‐point Likert scale, with higher values indicating more positive evaluations.

Reasons for Calling CIT. Participants rated the relevance of various reasons for contacting the CIT (Table 4). The highest mean ratings were given to stress experienced by patients' relatives, followed by patients' own stress and situations exceeding staff competencies.

**TABLE 4:** Requesters' reasons for contacting the Crisis Intervention Team (CIT).

Reason	M	SD
Patient's stress	4.21	0.92
Patient family's stress	4.71	0.50
Staff's time constraints	3.65	1.26
Situations exceeding staff competencies	4.00	1.15
Personal stress	3.33	1.29
Staff's adoption of patient's emotions	2.98	1.36

*Note:* Ratings were based on a 5‐point Likert scale, with higher values indicating stronger perceived relevance of the reason.

Abbreviations: M, Mean; SD, Standard Deviation.

#### 3.2.3 Group Comparisons of Perceived Work Characteristics (Quantitative Workload, Autonomy, Role Clarity)

Quantitative workload did not differ significantly between CIT‐requesters (M = 19.13, SD = 5.88) and Non‐requesters (M = 18.55, SD = 5.67), t(68) = 0.39, *p* = 0.700, *d* = 0.10, 95% CI [−0.41, 0.60].

Autonomy also did not differ significantly between CIT‐requesters (Md = 21.5, IQR = 6.0) and Non‐requesters (Md = 22.5, IQR = 8.25), *U* = 519.00, Z = −0.11, *p* = 0.909, *r* = 0.01.

In contrast, role clarity was significantly higher in CIT‐requesters (Md = 21.0, IQR = 4.0) than in Non‐requesters (Md = 20.0, IQR = 6.25), *U* = 352.00, Z = −2.24, *p* = 0.025, *r* = 0.27, indicating a small‐to‐medium effect. The Hodges–Lehmann estimate of the between‐group difference was 2.00, 95% CI [0.00, 4.00] (Table 5).

**TABLE 5:** Between‐group comparisons of perceived work characteristics (requesters vs. non‐requesters).

Variable	Group	M	SD	Md	IQR	Test statistic (*t*‐test/Mann–Whitney *U*‐test)	*p*	Effect size (Cohen's *d*/*r*)
Quantitative workload	Requesters	19.13	5.88	18.5	10.0	*t*(68) = 0.39	0.700	*d* = 0.10 (95% CI [−0.41, 0.60])
	Non‐requesters	18.55	5.67	18.5	9.0			
Autonomy	Requesters	21.33	4.45	21.5	6.0	*U* = 519.00, Z = −0.11	0.91	*r* ≈0.01
	Non‐requesters	21.09	5.66	22.5	8.25			
Role clarity	Requesters	20.65	3.18	21.0	4.0	*U* = 352.00, Z = −2.24	0.025*	*r* ≈0.27
	Non‐requesters	18.36	3.96	20.0	6.25			

*Note:* Workload was normally distributed and analysed with an independent samples *t*‐test; values are reported as M (SD) together with Cohen's *d* and its 95% confidence interval. Autonomy and Role Clarity showed non‐normal distributions and were therefore analysed with Mann–Whitney *U*‐tests; values are reported as Md (IQR) together with effect size *r* (calculated as |*Z*|/√*N*). According to Cohen [40], *r* = 0.10 indicates a small, *r* = 0.30 a medium, and *r* = 0.50 a large effect. An asterisk (*) indicates statistical significance at *p* < 0.05. For role clarity, the Hodges–Lehmann estimate of the between‐group difference was 2.00, 95% CI [0.00, 4.00].

Abbreviations: IQR, interquartile range; M, mean; Md, median; SD, standard deviation.

#### 3.2.4 Moderation Analysis

Four linear regression moderation analyses examined whether age, job tenure, supervisor support and colleague support influenced the association between CIT exposure and the total PWCS score.

Age did not moderate the association between CIT exposure and the PWCS total score, Δ*R* ^2^ < 0.001, *F*(1,66) = 0.004, *p* = 0.949. A reduced regression model without the interaction term was significant, *R* ^2^ = 0.087, *F*(2,67) = 3.19, *p* = 0.047. Age was positively associated with the PWCS total score, B = 2.87, SE = 1.29, *β* = 0.26, *t* = 2.22, *p* = 0.030, 95% CI [0.29, 5.45], indicating that younger employees reported lower scores and therefore greater perceived strain. CIT exposure showed no significant main effect in this model, B = 2.38, SE = 2.59, *t* = 0.92, *p* = 0.362, 95% CI [−2.79, 7.54].

Job tenure neither showed a significant main effect on the PWCS total score, B = −0.64, SE = 1.74, *t* = −0.37, *p* = 0.713, 95% CI [−4.10, 2.82], nor significantly moderated the association between CIT exposure and PWCS scores, B = 2.80, SE = 2.14, *t* = 1.31, *p* = 0.196, 95% CI [−1.48, 7.08], Δ*R* ^2^ = 0.024.

Supervisor support significantly moderated the association between CIT exposure and the PWCS total score, B = −5.23, SE = 2.14, *t* = −2.44, *p* = 0.017, 95% CI [−9.50, −0.95], Δ*R* ^2^ = 0.073. At low levels of supervisor support, CIT‐requesters reported significantly higher PWCS scores than non‐exposed staff, B = 10.33, SE = 4.14, *t* = 2.49, *p* = 0.015, 95% CI [2.06, 18.61], whereas no significant group differences were observed at moderate or high levels of supervisor support (Figure 2).

**FIGURE 2:**
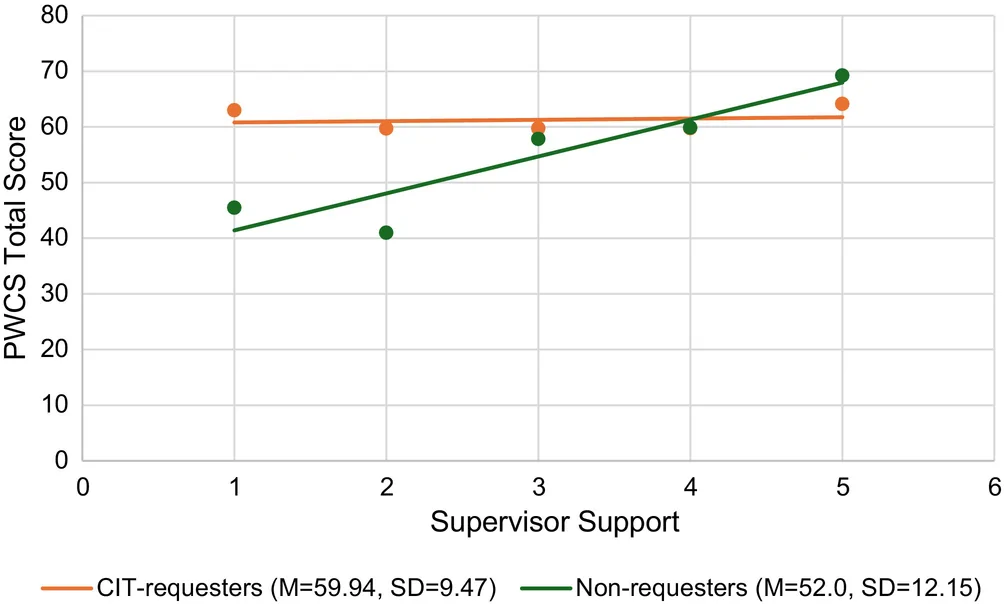
Interaction effect of supervisor support and CIT‐request on perceived work characteristics (PWCS Total Score). Non‐requesters with low supervisory support reported the highest levels of strain. Higher PWCS Total Scores indicate more favourable perceived work characteristics.

Colleague support did not significantly moderate the association between CIT exposure and PWCS scores, B = 5.60, SE = 3.66, *t* = 1.53, *p* = 0.130, 95% CI [−1.70, 12.91], Δ*R* ^2^ = 0.031. However, in a reduced model without the interaction term, colleague support was positively associated with the PWCS total score, B = 4.27, SE = 1.83, *β* = 0.28, *t* = 2.33, *p* = 0.023, 95% CI [0.62, 7.92].

Interaction effects and significant main effects from the reduced models are summarised in Table 6.

**TABLE 6:** Moderation analyses on total PWCS score.

Moderator	Interaction B	SE	*t*	*p*	95% CI	Δ*R* ^2^	Significant main effect in reduced model
Age	−0.18	2.71	−0.06	0.949	[−5.59, 5.24]	< 0.001	Age: B = 2.87, SE = 1.29, *β* = 0.26, *t* = 2.22, *p* = 0.030, 95% CI [0.29, 5.45]
Tenure	2.80	2.14	1.31	0.196	[−1.48, 7.08]	0.024	None
Supervisor support	−5.23	2.14	−2.44	0.017	[−9.50, −0.95]	0.073	No main effect; significant interaction
Colleague support	5.60	3.66	1.53	0.130	[−1.70, 12.91]	0.031	Colleague support: B = 4.27, SE = 1.83, *β* = 0.28, *t* = 2.33, *p* = 0.023, 95% CI [0.62, 7.92]

*Note:* Main effects for age and colleague support were obtained from reduced models without the interaction term.

Abbreviations: Δ*R* ^2^, change in explained variance; CI, confidence interval; PWCS, Perceived Work Characteristics Scale.

## 4 Study II: Focus Groups

### 4.1 Design and Methods

#### 4.1.1 Recruitment

For the qualitative phase, two profession‐specific focus groups with 5–7 participants each were planned a priori. This approach is supported by empirical evidence suggesting that most themes can be identified within two to three focus groups in relatively homogeneous samples [41]. As discussions followed pre‐defined topics rather than an open exploration, thematic saturation was expected to be reached within two sessions. Recruitment took place through personal contact to ensure all participants had prior experience with at least one CIT intervention. The focus groups were conducted in August 2024. Written informed consent was obtained at the beginning of each focus group session. While no financial incentives were provided, participants received a hospital cafeteria voucher as a token of appreciation.

#### 4.1.2 Data Analysis

The qualitative phase was designed to address unresolved questions from the survey and to deepen the understanding of key themes identified in the quantitative data. To explore these issues, a semi‐structured focus group guide was developed (Appendix S4). In line with the explanatory sequential design, four main themes were defined deductively based on the quantitative findings and study objectives, and one additional main theme as well as several subcategories were derived inductively from the data. The material was analysed using Mayring's qualitative content analysis. Two researchers coded independently and resolved discrepancies by consensus [42].

#### 4.1.3 Qualitative Rigour and Reflexivity

To ensure rigour in Study II, we drew on Lincoln and Guba's criteria of trustworthiness for qualitative research, including credibility, transferability, dependability and confirmability [43]. Credibility was supported through independent coding by two researchers and consensus discussions. Dependability was enhanced by documenting analytic decisions, and confirmability was ensured by grounding all interpretations in the data. Transferability was facilitated by providing contextual information about participants and the clinical setting. These procedures were consistent with the MMAT criteria, as they supported transparency and coherence across the qualitative approach, data collection, analysis, and interpretation.

Reflexivity was maintained throughout the qualitative phase. The researcher who conducted and analysed both study components had professional experience in psychosocial care but was not a member of the hospital's CIT. The broader research team included both CIT and non‐CIT members; this heterogeneity was used intentionally to discuss potential preconceptions, bracket assumptions, and minimise the influence of individual researcher perspectives on theme development. To avoid influencing the discussion process, a CIT member joined the focus groups only during the final minutes to address factual questions raised by participants, ensuring that the data collection itself remained non‐interventionist.

### 4.2 Findings

#### 4.2.1 Characteristics of the Participants

Focus Group one consisted of five physicians (one female, four male) working in ICU and ED at a large university hospital in Germany. Focus Group two included seven female nurses and midwives from paediatric ICU and neonatology. To maintain participant anonymity, no further demographic details were collected.

#### 4.2.2 Staff Experiences With CIT

The overarching categories ‘Reasons for request’ (f = 49), ‘Barriers for request’ (f = 35), ‘Suggestions for improvement’ (f = 29) and ‘CIT as supportive resource’ (f = 40) were defined a priori using a deductive approach. An additional overarching category, ‘Questions/Need for information’ (f = 9), was identified inductively during the qualitative content analysis. To enhance transparency of the analytic process, a coding tree illustrating the development of themes from raw data to higher‐order categories is provided in Table S2. Table S3 provides an overview of the codes contained in the complete category system and their frequency across the focus group interviews. Here and in the following, f refers to the number of coded statements assigned to a category.

Reasons for request. The most frequently cited reason was the presence of highly distressed relatives (f = 13), in line with the survey findings. Another frequently mentioned reason involved cases with children and young adults (f = 6), described as emotionally charged situations that heightened staff vulnerability, thereby increasing the perceived need for psychosocial support.

Acute clinical deterioration (f = 5) was among the five most frequently cited reasons for involving the CIT, alongside ‘Out‐of‐hours crisis’ and therefore the absence of an on‐site ward psychologist (f = 5), which was perceived as a structural gap in immediate support. Situations that exceeded staff's professional competencies (f = 5) were also highlighted as triggers for CIT involvement.

As one participant explained: ‘[I contact the CIT when] our way of communicating no longer succeeds in restoring balance, in accessing resources for coping with grief, or in drawing on inner capacities—in other words, when we are no longer making progress.’ (Focus Group 1, Pos. 16).

Barriers for request. The category ‘Barriers for request’ revealed several factors contributing to participants' hesitation in contacting the CIT. The most common reasons were urgent or unexpected situations (f = 7). A frequently mentioned concern was the fear of making an unnecessary request (f = 6), such as misjudging the severity of a situation and disturbing CIT members during off‐hours. As one participant explained:

I'd actually hesitate to make the call and say: it might get out of hand. (Focus Group 1, Pos. 85)

This quote illustrates a broader ambivalence about when a situation is considered ‘critical enough’ to warrant intervention—suggesting that the threshold for activation may be perceived as unclear. In addition, anticipated rejection by patients (f = 4) was mentioned as another barrier, particularly in emotionally sensitive or unpredictable encounters. Together, these findings point to perceptual barriers that may inadvertently discourage staff from requesting psychosocial support, even when it could be beneficial for patients and families.

Suggestions for improvement. Participants offered a total of 29 suggestions to enhance CIT usability and effectiveness. The most frequent recommendation concerned improved integration of CIT contact information into daily workflows, particularly embedding CIT details and availability in the hospital intranet (f = 4), whereas physicians favoured more immediate solutions, such as assigning the CIT to a speed‐dial key on hospital phones. Physicians also proposed further steps to strengthen interdisciplinary collaboration, including structured feedback after CIT involvement (f = 2) and a screening tool to identify at‐risk individuals earlier (f = 3), thereby enabling more systematic prioritisation of support needs.

Questions/Need for information. A further category of information needs emerged, particularly among physicians, centring on role clarity, availability and accessibility of the CIT. Limited on‐call hours were frequently questioned, raising doubts about the appropriateness of the term crisis intervention for a service with restricted temporal access. Uncertainty about availability created hesitation, particularly in borderline or time‐sensitive situations. As one physician noted: ‘So, to put it bluntly: if the crisis happens on a Friday, I won't call you, but if it happens on a Saturday, I will?’ (Focus Group 1, Pos. 57).

This illustrates the confusion caused by non‐continuous availability and the mismatch between the urgency of events and organisational accessibility. The same participant later summarised this tension succinctly: ‘It's more complicated than I would like it to be.’ (Focus Group 1, Pos. 65).

CIT as supportive resource. A total of 40 statements were coded under ‘CIT as supportive resource’, 16 of which referred to personal emotional relief. Both physicians and nurses indicated that CIT facilitated creating distance from the situation. Physicians often emphasised their emotional detachment from the case from the outset and described the CIT as an additional buffer between themselves and the patient. Nurses, in contrast, drew on the CIT more directly for emotional relief, citing a wider variety of supportive effects, as a ‘calming presence’ and reassurance that help was ‘on the way’.

Beyond personal emotional relief, participants also highlighted other supportive functions of the CIT, most often support for patients' relatives (f = 9), followed by time relief for staff (f = 7). Physicians noted, however, that any time gained was quickly absorbed by other duties, preventing a genuine reduction in workload.

## 5 Integration of Findings

The qualitative findings contextualised the quantitative results by indicating that CIT involvement clarified psychosocial and clinical responsibilities without changing formal autonomy. Temporary time relief was quickly absorbed by other duties, helping to explain the absence of lower overall workload. The qualitative data did not directly explain the supervisor‐support interaction. However, participants' references to organisational permission and leadership endorsement provided limited contextual support for interpreting this quantitative finding.

## 6 Discussion

Study I showed that CIT support was evaluated positively, although limited on‐call availability remained a major concern. CIT‐requesters reported greater role clarity, whereas no group differences were found for workload or autonomy; supervisor support also emerged as an important contextual factor. Study II contextualised these findings, with the integration of both phases summarised in a joint display in Appendix S5.

The qualitative findings helped explain the quantitative results in several ways.

The higher role clarity among CIT‐experienced staff was explained by participants‘ descriptions of the CIT assuming psychosocial and communicative tasks, thereby clarifying staff responsibilities during acute situations. The absence of a measurable reduction in workload was explained by reports that temporary time relief was quickly absorbed by other clinical duties. Similarly, the lack of differences in autonomy was consistent with participants‘ accounts of the CIT as a supportive resource that did not alter formal decision‐making authority, professional hierarchies, or clinical protocols.

Taken together, the integration of both study phases showed that CIT function as a situational and context‐dependent organisational resource: they clarify responsibilities and provide psychosocial relief during acute crises, but do not alter formal autonomy or the structural conditions underlying overall workload.

Study II further identified distressed relatives, complex paediatric cases and situations exceeding professional competencies as common reasons for requesting CIT support. This aligns with findings from a US intensive care study showing that emotional strain among patients and relatives is closely linked to increased stress among nursing staff [44]. The focus groups also identified unclear activation thresholds, concerns about overburdening the CIT, and profession‐specific differences in perceived emotional relief as important contextual factors shaping CIT use in practice.

Workload, Autonomy and Role Clarity. In line with the JD‐R model, CIT can be regarded as a secondary resource buffering acute psychosocial strain. The absence of measurable effects on workload and autonomy in Study I calls for closer examination. Several mechanisms may account for these findings.

First, CIT provide situational support rather than structural changes in task volume or decision latitude. CIT may nevertheless reduce task‐switching demands and related negative consequences, such as failing to resume suspended tasks, as observed in an Australian emergency department study [45].

Second, autonomy in hospitals is strongly shaped by professional hierarchies and institutional protocols, with substantial variability across professions and clinical settings. For instance, a Finnish study found that nurses reported more autonomy in direct patient care than in unit‐level decisions [46]. This underscores the limited structural influence of CIT compared with systemic approaches such as shared leadership, which an integrative review identified as potentially supporting professional autonomy in nursing [47]. By contrast, the higher role clarity observed in Study I suggests that acute interventions can provide orientation in moments of crisis, complementing—but not transforming—existing organisational frameworks.

Third, the absence of a group difference in autonomy may also reflect a plateau effect. According to Warr‘s vitamin model, additional autonomy may provide little further benefit once an adequate level has been reached [48, 49]. However, as baseline autonomy levels and non‐linear associations were not specifically examined, this interpretation remains tentative.

Supervisor Support. Staff without prior CIT involvement reported the highest strain when supervisor support was low, suggesting that CIT may provide situational support when other organisational resources are limited. However, sustainable strain reduction is likely to require a combination of situational interventions and broader organisational resources, consistent with evidence from international systematic reviews of organisational and workplace mental health interventions [50, 51].

Structural Needs. Across both studies, limited CIT availability emerged as a central concern. Participants expressed frustration about limited accessibility, particularly the absence of 24/7 coverage and uncertainty about when and how to initiate a request. By contrast, the team‘s credibility, relevance and utility in crisis situations were consistently affirmed. Together with recommendations for family‐centred ICU care [52], these findings support the organisational integration of professional psychosocial support in high‐pressure settings. At the same time, caution is warranted in light of the ‘waking up sleeping dogs’ effect, whereby drawing attention to certain psychosocial issues may inadvertently heighten dissatisfaction that might otherwise have remained less salient [53].

Impact on Strain. In Study II, a noteworthy pattern concerned differences between physicians and nurses: Physicians often emphasised support for relatives as a key benefit of CIT involvement, whereas nurses highlighted their own need for emotional relief. For physicians, this may indicate emotional distancing that helps sustain effective performance under pressure. Nurses, by contrast, are continuously engaged in bedside care, making such distancing more difficult. This interpretation aligns with findings from a German study in which emotional exhaustion was the strongest predictor of ‘cool down’ reactions (emotional distancing as an adaptive strategy to maintain functionality at work) among nurses, while depersonalization was most relevant for physicians [54]. Taken together, these findings suggest that profession‐specific role demands may influence how staff benefit from CIT involvement. At the same time, it is important to acknowledge that crises constitute only a fraction of daily work, while many other demands, such as documentation, occur independently. Overemphasising crises may therefore exaggerate their contribution to overall occupational strain.

## 7 Limitations

When interpreting the results, several limitations should be taken into account. First, the use of convenience sampling at a single university hospital limits representativeness and increases the risk of self‐selection bias. Staff with more favourable attitudes towards the CIT may have been overrepresented, while critical perspectives were underrepresented. This also applies to Study II, as the focus groups may have been dominated by participants with predominantly positive experiences, thereby restricting the generalisability of the findings. In addition, data collection in a high‐demand clinical environment further increased the likelihood of premature dropout.

Second, the study relied on retrospective self‐reports, which are subject to recall bias. In Study I, some crises occurred up to 2 years prior, and CIT‐requesters and Non‐requesters may not have reflected events of comparable severity. This could have produced less favourable baseline conditions for the CIT‐requesters and contributed to converging group means.

Third, although the PWCS provided useful insights in Study I, its suitability for capturing acute intervention effects is uncertain. The instrument was translated without full psychometric validation, and the modest sample size reduced statistical power and generalisability. Moreover, strain assessment was limited to self‐reports and not complemented by behavioural or physiological indicators.

Fourth, no a priori sample size calculation was conducted. Given the exploratory, field‐based design, the sample comprised all eligible staff available during the data collection period rather than a predetermined target. While appropriate for this context, this limits statistical power and increases the risk of Type II errors, meaning that some non‐significant findings—particularly for workload and autonomy—should be interpreted with caution.

Finally, the mixed methods design developed iteratively rather than being fully specified prospectively. Although the qualitative phase was deliberately built on questions emerging from Study I, the time interval between phases and the inclusion of only CIT‐experienced staff in Study II limited direct comparison with Non‐requesters. The qualitative findings therefore contextualise plausible mechanisms and experiences but cannot establish why the quantitative group differences occurred at the individual participant level.

## 8 Recommendations for Practice

The findings of this study suggest several implications for clinical practice in high‐acuity settings, particularly for critical care nurses working in ICU and ED settings. Given their continuous bedside presence and close involvement with patients and relatives, nurses are frequently exposed to intense emotional demands, especially in situations involving distressed relatives, paediatric emergencies or sudden clinical deterioration.

CIT may serve as a valuable support resource for critical care nurses by providing immediate psychosocial care for patients and relatives, thereby allowing nurses to step back emotionally and refocus on essential clinical tasks. Early and nurse‐initiated activation of the CIT may help prevent cumulative emotional strain and reduce role conflict in acute situations.

The findings further indicate that uncertainty about activation thresholds represents a key barrier. Clear, nurse‐oriented activation criteria, visible integration of CIT contact information into daily workflows (e.g., intranet access or emergency phone lists) and explicit endorsement of CIT use by leadership may lower barriers to utilisation.

Although CIT are available to all hospital staff, the results highlight that nurses in critical care settings experience particularly strong emotional relief from CIT involvement due to their sustained proximity to patients and relatives. Integrating information about CIT into nursing onboarding, simulation training and post‐event debriefings may therefore strengthen nurses' confidence in using this resource effectively.

## 9 Conclusion

This study provides one of the first systematic evaluations of CIT in hospitals from the staff perspective. CIT are perceived as a valuable organisational resource that enhances role clarity and offers psychosocial relief in emotionally demanding contexts, particularly in ICU and ED settings. However, their effectiveness depends on complementary resources such as supervisory support, while structural barriers—especially limited availability—remain major obstacles. If sustainably integrated and supported by leadership, CIT can improve both staff well‐being and patient care. Because early encounters strongly shape perceptions, providers have only a narrow window to establish trust and credibility.

## Funding

The authors have nothing to report.

## Ethics Statement

Study I was approved by the Ethics Committee of Jena University Hospital on 11 April 2023 (Ref. No. 2023‐2974‐Bef). Study II received separate approval from the same committee on 11 June 2024 (Ref. No. 2024‐3394‐Bef).

## Consent

The authors have nothing to report. No patients were included in this study. Written or electronic informed consent was obtained from all participating healthcare professionals.

## Conflicts of Interest

The authors declare no conflicts of interest.

## Supporting information


**Table S1:** Overview of variables assessed in the survey and focus groups, respondent groups and analytical approaches.

**Table S2:** Coding tree illustrating the development of qualitative themes.

**Table S3:** Category system.

**Appendix S1:** MMAT assessment.

**Appendix S2:** Good reporting of a mixed methods study (GRAMMS) checklist [55].

**Appendix S3:** Evaluation questionnaire (translated).

**Appendix S4:** Focus group guide (translated).

**Appendix S5:** Joint display integrating quantitative and qualitative findings in an explanatory sequential mixed methods design.

## Data Availability

The datasets generated and/or analysed during the current study are not publicly available due to institutional and ethical restrictions but are available from the corresponding author upon reasonable request.
