# Supplementary material for: Crisis Intervention Teams as Dual Resources: An Explanatory Sequential Mixed Methods Study of Hospital Staff
Source: Nurs Crit Care. 2026 Nov 10;31(6):e70692. doi: 10.1111/nicc.70692 (PMC13752548; doi:10.1111/nicc.70692)
Supplement: Supplementary file 1 [file NICC-31-0-s001.docx]

Supplementary Table S2. Coding tree illustrating the development of qualitative themes

| Raw Data | Inductive Code | Sub-subcategory | Subcategory | Higher-order category | Deduktiv/Induktiv |
| --- | --- | --- | --- | --- | --- |
| “I also recall a similar case in which the crisis intervention team was involved. In that situation, however, the father was experiencing severe psychological distress and was struggling significantly to cope with the situation.” | Severe psychological distress of relatives | / | Distressed relatives | Reasons for request | The main category was defined deductively. Subcategories were derived inductively. |
| “And sometimes there are cases that completely overwhelm you—when a child requires resuscitation in the emergency department and you’re transporting them to the ICU while ongoing resuscitation is still in progress. You’re so caught up in what you’re doing that you completely lose sight of the relatives.” | Overwhelming acute situations | / | Unexpected cases | Barriers for request | The main category was defined deductively. Subcategories were derived inductively. |
| “What also comes to mind is that on the intranet page […] the crisis intervention team could be placed there as well. […] That’s a place people tend to check more often, for example when they’re looking for something or want to access Outlook, and then they would see: crisis intervention team.” | Limited visibility of the crisis intervention team | Integration into intranet | Improvement suggestions for the CIT | Suggestions for improvement | The main category was defined deductively. Subcategories were derived inductively. |
| “For me, that clearly represents a relief in that situation. I take a step back. I’m no longer quite as closely involved with the woman and the family.” | Emotional distancing as relief | / | Own emotional relief | CIT as supportive resource | The main category was defined deductively. Subcategories were derived inductively. |
| “Is the crisis intervention team actually almost always called for relatives, or sometimes also for patients?” | Uncertainty about target group of the crisis intervention team | / | Responsibilities | Questions / Need for information | The main category was defined inductively. Subcategories were derived inductively. |

**Note**. This coding tree illustrates the analytic process from raw data to higher-order categories, including inductively derived codes, subcategories, and sub-subcategories, and their integration into overarching themes, using illustrative examples of the analytic process. Higher-order categories were defined deductively to address gaps identified in the preceding quantitative study, whereas subcategories and sub-subcategories were derived inductively from the qualitative material. The category “Questions / need for information” emerged inductively based on questions raised during the focus groups. The complete category system is reported separately.
