# Supplementary material for: Crisis Intervention Teams as Dual Resources: An Explanatory Sequential Mixed Methods Study of Hospital Staff
Source: Nurs Crit Care. 2026 Nov 10;31(6):e70692. doi: 10.1111/nicc.70692 (PMC13752548; doi:10.1111/nicc.70692)
Supplement: Supplementary file 2 [file NICC-31-0-s003.docx]

Supplementary Appendix S3 – Evaluation Questionnaire (translated)

**Work Characteristics during the Crisis Situation supplemented by support**

(Based on the Perceived Work Characteristics Survey, Haynes et al., 1999)

Response scale for autonomy, work demands and role clarity: 1 = not at all, 2 = just a little, 3 = moderate amount, 4 = quite a lot, 5 = a great deal

*Autonomy*

“To what extend do you…?”

I. Determine the methods and procedures you use in your work?
II. Choose what work you will carry out?
III. Decide when to take a break?
IV. Vary how you do your work?
V. Plan your own work?
VI. Carry out your work in the way you think best?

*Work Demands*

“How often do you find yourself meeting the following problems in carrying out your work in acute crisis?”

I. I do not have enough time to carry out my work.
II. I cannot meet all the conflicting demands made on my time at work.
III. I never finish work feeling I have completed everything I should.
IV. I am asked to do work without adequate resources to complete it.
V. I can not follow best practice in the time available.
VI. I am required to do basic tasks which prevent me completing more important ones.

*Role clarity*

“How true are the following statements for your work in acute crisis?”

I. I have clear planned goals and objectives for my work.
II. I know that I have divided my time properly.
III. I know what my responsibilities are.
IV. Explanation is clear of what has to be done.
V. I know exactly what is expected of me.

*Support*

“How true are the following statements for your work in acute crisis?”

I. I could rely on support from my supervisor.
II. I could rely on support from my team colleagues.

**Evaluation of the crisis intervention**

“When I contacted the CIT…”

1 I was able to reach them on the first attempt.
2 I had to make several attempts before I could reach them.
3 I was not the person who contacted the CIT.

“The CIT was available with the urgency I required (e.g. immediately, same day):”

1 Available
2 Not available
3 Other
4 I was not the person who made the contact

“The current on-call hours of the CIT (Sat/Sun/holidays 07:00–21:00) are, in my view …”

1 Sufficient
2 Not sufficient

If 2: “Please indicate the on-call hours you would consider optimal: [Open text]”

“The responsibilities and tasks of the CIT are …”

1 Not known at all
2 Rather unknown
3 Partially known
4 Rather known
5 Fully known
6 Cannot assess

“After the CIT deployment, I was …”

1 Not informed sufficiently
2 Rather not informed sufficiently
3 Partially informed sufficiently
4 Rather informed sufficiently
5 Informed sufficiently
6 Cannot assess

“Agreements with the CIT were …”

1 Not binding
2 Rather not binding
3 Partially binding
4 Rather binding
5 Binding
6 Cannot assess

“Through the availability of the CIT …”

1 I have greater scope of action with patients/relatives in crisis.
2 I experienced no change in my scope of action.
3 I experienced a reduction in my scope of action.

“Overall, I perceive the CIT as …”

1 An additional burden
2 No change
3 An additional resource
4 Other [Open text]

**Connection Between CIT and Own Experience**

“I contact the CIT when …” (Likert scale 1–5)

I. I perceive strong emotional distress in the patient.
II. I perceive strong emotional distress in the relatives of the patient.
III. My time resources are insufficient to devote to the case as needed.
IV. I anticipate that the case exceeds my own competence and experience.
V. I experience or expect strong emotional distress in myself due to the case.
VI. I notice that I adopt the patient’s crisis-related feelings (“standing in the patient’s shoes”).

**Open text questions**

“ What was positive about the CIT deployment(s)? [Open text]”

“What should be improved? [Open text]”
