# Supplementary material for: Crisis Intervention Teams as Dual Resources: An Explanatory Sequential Mixed Methods Study of Hospital Staff
Source: Nurs Crit Care. 2026 Nov 10;31(6):e70692. doi: 10.1111/nicc.70692 (PMC13752548; doi:10.1111/nicc.70692)
Supplement: Supplementary file 3 [file NICC-31-0-s004.docx]

Supplementary Appendix S2 – Good Reporting of a Mixed Methods Study (GRAMMS) Checklist (O’Cathain et al., 2008)

| No. | GRAMMS criterion | Location in current manuscript | Assessment | Comments |
| --- | --- | --- | --- | --- |
| 1 | Describe the justification for using a mixed-methods approach to the research question. | Abstract – Aim and Study design; Introduction – Aim; General Design & Methods – Research Design Overview | Reported | The manuscript explains that the quantitative survey identified patterns and unresolved questions, whereas the subsequent focus groups were used to explain and contextualize those findings. This rationale is consistent with an explanatory sequential mixed-methods approach. |
| 2 | Describe the design in terms of the purpose, priority, and sequence of methods. | Abstract – Study design; Introduction – Aim; General Design & Methods – Research Design Overview | Reported | The quantitative survey is identified as the first and prioritized phase, followed by two profession-specific focus groups. The qualitative phase is explicitly described as explaining and contextualizing the quantitative findings. |
| 3 | Describe each method in terms of sampling, data collection, and analysis. | Study I Survey – Recruitment and Study Design, Measures, Procedure, Data analysis; Study II Focus Groups – Recruitment, Data analysis, Qualitative Rigor and Reflexivity | Reported | Sampling, eligibility, recruitment, data collection, measures, and analysis are described separately for both phases. The final samples are reported as N = 70 for Study I and N = 12 for Study II. |
| 4 | Describe where integration occurred, how integration occurred, and who was involved in integration. | General Design & Methods – Research Design Overview; Study II – Data analysis and Reflexivity; Study II Results – Integration of findings; Discussion; Appendix E (joint display) | Reported | The manuscript identifies building, connecting, and merging as integration strategies. Study I findings informed the focus-group guide and the selection of CIT-experienced participants. Two researchers independently coded the qualitative data and resolved discrepancies by consensus, while the broader research team discussed interpretations. Integration is made explicit in the Results, Discussion, and joint display. |
| 5 | Describe any limitation of one method associated with the presence of the other method. | Limitations – final paragraph | Reported | The manuscript states that the mixed-methods design developed iteratively rather than being fully specified prospectively. It addresses the interval between phases, the inclusion of only CIT-experienced staff in Study II, the resulting limitation in direct comparison with Non-requesters, and the inability to establish individual-level explanations for the quantitative findings. |
| 6 | Describe any insights gained from mixing or integrating methods. | Study II Results – Integration of findings; Discussion; Recommendations for practice; Conclusion; Appendix E (joint display) | Reported | The manuscript explains how the qualitative findings contextualized greater role clarity and the absence of differences in workload and autonomy. It also states the added value of integration by characterizing CIT as a situational and context-dependent organizational resource that provides psychosocial relief and clarifies responsibilities without altering formal autonomy or structural workload conditions. |

Overall assessment. All six GRAMMS reporting criteria are addressed in the current manuscript. The qualitative phase was added iteratively after the quantitative study. Accordingly, GRAMMS was not used prospectively to design the overall mixed-methods study. The checklist was completed retrospectively to assess and improve the transparency and completeness of reporting in the revised manuscript.
