# Supplementary material for: Crisis Intervention Teams as Dual Resources: An Explanatory Sequential Mixed Methods Study of Hospital Staff
Source: Nurs Crit Care. 2026 Nov 10;31(6):e70692. doi: 10.1111/nicc.70692 (PMC13752548; doi:10.1111/nicc.70692)
Supplement: Supplementary file 4 [file NICC-31-0-s005.docx]

Supplemetary Table S1. Overview of variables assessed in the survey and focus groups, respondent groups and analytical approaches

| **Category** | **Subcategory/Item** | **Source** | **Requesters** | **Non-requesters** | **Analysis Method** |
| --- | --- | --- | --- | --- | --- |
| Sociodemographic/ occupational | - Gender - Frequency of crisis situations on the respondent’s home ward - Percentage indicating how often support was received and how often it was desired - Ward on which the CIT intervention took place - Year of the CIT intervention - Whether the CIT was requested by the respondent themselves or by a colleague | Survey | ✓ | Only gender | Descriptive analysis |
| Moderators | - Age - Years of experience - Supervisor support - Colleague support | Survey | ✓ | ✓ | Linear regression |
| Structural and Procedural Aspects | - Reachability of the CIT - Availability of the CIT - Sufficiency of on-call hours of the CIT - Clarity of responsibilities of the CIT - Effectiveness of information flow between the requester and the CIT - Reliability of the CIT | Survey | ✓ |  | Descriptive analysis |
| Reasons for Call | - Patient’s stress - Patients’ family’s stress - Staff’s time constraints - Staff’s exceeding competencies - Staff’s stress - Staff’s adoption of patient’s emotions | Survey | ✓ |  | Descriptive analysis |
| Others | - Perceived helpfulness for the affected individuals from staff’s perspective - Improvement suggestions | Survey | ✓ |  | Descriptive analysis |
| Perceived Work Characteristics | - Autonomy - Quantitative workload - Role clarity | Survey | ✓ | ✓ | T-test for quantitative workload; Mann–Whitney U-test for autonomy and role clarity due to unmet requirements |
| Further experiences with CIT | - Questions / Need for information - CIT as supportive resource - Suggestions for improvement - Barriers for request - Reasons for request | Focus groups | ✓ |  | Mayring’s qualitative content analysis |

**Note.** This table provides an overview of all variables assessed across the quantitative survey and qualitative focus groups, including the data source, respondent group(s), and analytical approach. A checkmark (✓) indicates that the respective variable was assessed (collected) in the indicated respondent group. Some variables were assessed only among Crisis Intervention Team (CIT) requesters, whereas perceived work characteristics and moderators were assessed in both requesters and non-requesters to allow group comparisons. Structural and procedural aspects and reasons for contact were analysed descriptively. Perceived work characteristics (autonomy, quantitative workload, and role clarity) were compared between groups: workload met normality assumptions and was tested using an independent-samples t test (Cohen’s d), whereas autonomy and role clarity violated normality assumptions and were analysed using Mann–Whitney U tests (effect size r). Moderation analyses were conducted to examine boundary conditions of CIT effects.
