# Supplementary material for: Crisis Intervention Teams as Dual Resources: An Explanatory Sequential Mixed Methods Study of Hospital Staff
Source: Nurs Crit Care. 2026 Nov 10;31(6):e70692. doi: 10.1111/nicc.70692 (PMC13752548; doi:10.1111/nicc.70692)
Supplement: Supplementary file 5 [file NICC-31-0-s007.docx]

Supplementary Appendix S5 - Joint Display Integrating Quantitative and Qualitative Findings in an Explanatory Sequential Mixed-Methods Design

| **Quantitative Finding** | **Qualitative Finding** | **Integrated Interpretation** |
| --- | --- | --- |
| Higher role clarity among CIT-requesters | The CIT provides orientation and guidance during crises, enabling healthcare staff to gain distance from the situation and concentrate on their medical tasks. | CIT enhances acute role clarity in crisis situations, explaining higher scores among requesters. |
| No differences in workload | Temporary relief from CIT is quickly absorbed by other duties; workload is structurally determined. | CIT reduces emotional/communicative load but cannot change structural workload. |
| No differences in autonomy | Accounts related to autonomy were mixed: one physician described reliance on nurses’ cues to recognise crises, suggesting a partial softening of hierarchical boundaries, while a nurse reported being prohibited by a superior from activating CIT, illustrating persistent hierarchical constraints. At the same time, participants emphasized that everyone is allowed to contact CIT, which conveys a sense of empowerment. | Because qualitative accounts point in both directions—limited softening of hierarchies in some situations, but reinforcement of hierarchy in others—no clear pattern emerges. This aligns with the quantitative result showing no group differences in autonomy. CIT provides situational empowerment by allowing anyone to activate support, but it does not meaningfully shift formal hierarchical structures. |
| Moderation: highest strain among non-requesters with low supervisory support | Supervisory support did not emerge as a theme in the qualitative analysis because the focus groups were structured around CIT-related experiences rather than broader organizational factors. | CIT serves as a secondary resource buffering strain when other supports are lacking. |
