# Supplementary material for: Crisis Intervention Teams as Dual Resources: An Explanatory Sequential Mixed Methods Study of Hospital Staff
Source: Nurs Crit Care. 2026 Nov 10;31(6):e70692. doi: 10.1111/nicc.70692 (PMC13752548; doi:10.1111/nicc.70692)
Supplement: Supplementary file 6 [file NICC-31-0-s008.docx]

Supplementary Appendix S4 – Focus Group Guide (translated)

*Reasons for requesting the CIT*

- What reasons lead you to request the Crisis Intervention Team (CIT)? Can you describe specific situations?

Follow-up questions:

- How do you determine whether a person is in crisis?
- Are there typical triggers or situations in which you tend to request the CIT more often?
- Could you describe a situation in which the presence of the CIT was particularly helpful?
- Could you describe a situation in which the presence of the CIT was not or less helpful?
- Are there certain times or shifts in which the CIT is needed more frequently?

*Reasons for not requesting the CIT*

- Can you describe situations in which you did not request the CIT, even though others might have? What kept you from doing so?

Follow-up questions:

- How do you determine whether a person is in crisis?
- Do you feel sufficiently informed about the options and services of the CIT? If not, what is lacking?
- Are there barriers or concerns that prevent you from using the CIT?
- What could we change to overcome these barriers?
- Have there ever been concerns about confidentiality or stigmatization when requesting the CIT?

*Framework conditions and suggestions for improvement*

- Which framework conditions or circumstances influence your decision to request the CIT?

Follow-up questions:

- Which organizational or structural factors make it easier or harder for you to request the CIT?
- What support and resources would you need to use the CIT more frequently or effectively?
- How could communication between you and the CIT be improved?
- Would training sessions or information events help you better understand and use the CIT?

*External influencing factors*

- How is it for you personally: does the CIT reduce your stress experience in crises, and why (or why not)?
